# Health care needs among recently arrived refugees in Germany: a cross-sectional, epidemiological study

**DOI:** 10.1007/s00038-020-01408-0

**Published:** 2020-09-17

**Authors:** Yuriy Nesterko, David Jäckle, Michael Friedrich, Laura Holzapfel, Heide Glaesmer

**Affiliations:** Department of Medical Psychology and Medical Sociology, University of Leipzig, Philipp-Rosenthal-Str. 55, 04103 Leipzig, Germany

**Keywords:** Refugees, Health care needs, PTSD, Depression, Somatization, Asylum

## Abstract

**Objectives:**

The purpose of the present study is to investigate current needs for physical and/or mental health treatment in recently arrived refugees’ by considering socio-demographic, flight, and mental health-related characteristics as well as different social care needs based on epidemiological data.

**Methods:**

The study was conducted in a reception facility for asylum-seekers in Leipzig, where 569 newly arrived adult residents participated. The questionnaire included socio-demographic and flight-related questions as well as standardized instruments for assessing mental health symptoms. Logistic regression models were conducted to predict current needs for treatment of self-rated physical and mental health status.

**Results:**

Greater numbers of traumatic events, positive screening results for at least one mental disorder, and a current need for assistance navigating the health care system were found to be significant predictors for current mental and physical health treatment needs. In addition, males are more likely to report current treatment needs for mental health symptoms.

**Conclusions:**

Health-related characteristics do predict newly arrived refugees’ treatment needs, and socio-demographic and flight-related characteristics do not. The results provide both academia and policy makers with first implications for improving health care for refugees in need as quickly as possible.

## Introduction

Migration rates are on the rise worldwide, a trend that has been gaining momentum over the last few decades in particular. There are many reasons to migrate, the most important being the desire for a better quality of life—in many cases caused by economic crises, political instability and/or armed conflicts currently present in many parts of the world. Besides voluntary migration, the number of forcibly displaced people seeking asylum has been dramatically growing in recent years. According to the Office of the United Nations High Commissioner for Refugees (UNHCR), by the end of 2018, 70.8 million people were forcibly displaced worldwide, including 25.9 million acknowledged refugees, and 3.5 million registered asylum-seekers, who were awaiting a decision on their asylum application, many of them in developing regions (United Nations High Commissioner for Refugees 2019).

By now, there is no doubt that the experiences people have while migrating impact their health status, even though the direction, type, and level of severity of this relationship are very complex and may consequently result in substantial health-related differences between different groups of immigrants (Nesterko et al. 2019). Especially forcibly displaced persons and/or refugees experiencing a broad spectrum of different adverse and/or stressful events before, during, and/or after fleeing have often been described as a high-risk population for developing mental disorders (Giacco et al. 2018), of which, post-traumatic stress disorder and depression have been the most frequently investigated (Bogic et al. 2015; Fazel et al. 2005; World Health Organization 2018). Thus, most high-income, Western countries are now dealing with increasing numbers of asylum-seekers, some of whom have complex health care needs and are consequently facing exceptional challenges addressing those needs appropriately within existing health care structures.

In general, available evidence on refugees’ health care needs is primarily focused on needs related to mental health problems (Giacco et al. 2018; Nesterko et al. 2020), as well as chronic (Goosen 2014) and infectious diseases (Yun et al. 2012). However, despite numerous reports indicating higher symptom burden, and in consequence increased needs for health care in different refugee populations, data on refugees’ health care needs and/or utilization remains inconsistent (Wetzke et al. 2018; Yang and Hwang 2016). This might be attributable to the great heterogeneity within refugee populations (e.g., due to countries of origin, flight route and duration, exposure to different traumatic events before, during, and/or after the flight) on the one hand, and differences in health policies towards asylum-seekers in different host countries on the other. In Germany, currently the leading destination for refugees and the country with the highest number of asylum applications in Europe, legal restrictions on access to health care were set up for asylum-seekers in 1993 (and were last revised in 2019). During the first 18 months after arrival, services including emergency medicine, care for acute and/or pain conditions, as well as pregnancy and child birth care are covered by the so-called Asylum-Seekers’ Benefits Act (AsylbLG). What is more, with the exception of emergencies, every health care utilization request made by asylum-seekers is subjected to review by the welfare agency staff after a personal application has been submitted by the asylum-seeker, a practice beholden to the subjective judgment procedure of local authority staff, who in many cases have no medical expertise and make their decisions based asylum-seekers’ subjective competence as well as language skills in applying for such services (Bozorgmehr et al. 2015). Consequently, language barriers and knowledge gaps concerning the health care system, both on the part of asylum-seekers and local authorities’ staff, lead to asylum-seekers experiencing restricted access to the health care system (Wångdahl et al. 2018), a state of affairs that has been widely criticized by researchers, health care providers, activists, and policy makers (Borgschulte et al. 2018). This applies in German federal states that reject electronic health cards for asylum seekers right after arrival, including Mecklenburg-Western Pomerania, Saxony-Anhalt, Hessen, Saarland, Baden-Wurttemberg, Bavaria and Saxony as well as parts of Lower Saxony, North Rhine-Westphalia and Rhineland-Palatinate. To the best of our knowledge, only six of 16 German federal states provide such cards for asylum seekers without restrictions (Medical care for refugees e.V. 2020).

In addition to the legal and bureaucratic barriers described above, there are factors on the individual level such as age and sex that influence health care needs and utilization in different refugee populations. In Germany, Wetzke et al. (2018) found higher healthcare utilization in refugee children age 10 and under as well as in refugees age 60 and older. Moreover, reports fairly consistently indicate that female refugees utilize health care more often than males do (Bozorgmehr et al. 2015; Gerritsen et al. 2006; Henjum et al. 2012). In their review on service utilization and barriers to accessing care for asylum-seekers, Hadgkiss and Renzaho (2014) identify and summarize some further factors more directly related to immigration, which may lead to reduced use of health care among refugees: poor health literacy and knowledge about the health care system in the host country, mistrust due to quality and confidentiality of care (e.g., quality of translation provided by interpreters, perceived connections between the immigration/asylum process and health systems, resulting in fear of possible deportation), educational, cultural and/or ethnic specifics in perception and expression of pain, as well as perceived discrimination by health care professionals. As a result, there are reports of over-utilization of emergency services occurring due to under-utilization of preventive and specialized health care services among various immigrant and refugee populations (Kiss et al. 2013; Sarría-Santamera et al. 2016), potentially also explained by the restrictions mentioned above. In general, the existing evidence reveals that, despite their higher level of needs (number of diagnoses, severity of symptoms, poor self-rated health status), asylum-seekers receive relatively few medical consultations and shorter durations of care compared to natives and other groups of immigrants (Bischoff and Denhaerynck 2010). This underscores the notion that access to health care does not automatically equal appropriate care (Newbold 2009). For example, in a population-based study Bidde et al. (2019) report about 30% of unmet health care needs stated by refugees and asylum seekers reflecting on the health care in Germany received during the last 12 months. In summary, different health care needs, resulting health care utilization, and existing barriers to the health care system among asylum-seekers should be seen as a complex interaction between the health care system, legal regulations, and the individual characteristics of refugee populations at risk.

Altogether, the existing evidence mentioned above primarily reflects the health care needs and health care utilization of immigrants and asylum-seekers, who had already been in their host country for a longer period of time. As a result, it reflects health care needs, which might be influenced by post-migration stressors (e.g., perceived discrimination, restricted access to the labor market, long-termed asylum procedure etc.). To the best of our knowledge, there are no studies available, which report about refugee’s health care needs upon their arrival in host countries. Therefore, the aim of the present study was to examine current needs for treatment of mental and physical health in recently arrived refugees. The analyses were conducted based on survey data collected in an epidemiological study, focusing on (1) socio-demographic and (2) flight-related characteristics, (3) mental health problems, as well as (4) different social care needs as potential predictors for self-rated current needs for treatment of physical and mental health in refugees of different origins upon their arrival in Germany.

## Methods

### Data collection and study sample

The study was conducted between May 2017 and June 2018 in a primary reception facility operated by the Federal State of Saxony for asylum-seekers in Leipzig, Germany. The study’s target population consisted of adult individuals (≥ 18 years) who were currently being accommodated in the facility during the survey period. Based on the facility’s registration data of all newly arrived residents, potential study participants were approached by members of the project staff in their accommodation unit, informed about the study objectives as well as data protection policy, and, in the event that they were willing to participate, introduced to the survey procedure. Between May 1st and May 15th of 2017, the participants were asked to fill out a paper version of the questionnaire (pilot study; *N* = 67), after May 17th of 2017, the participants filled out a tablet-based questionnaire in their respective native language. After information sheets and consent were handed out and consent to participate was given, the participants responded to the questionnaire by themselves (time needed approximately: 45 min). Project staff was available to answer questions when necessary. The assessments took place three times a week, on Mondays, Wednesdays, and Thursdays between 10 a.m. and 1 p.m. Data were electronically transferred and administered consecutively to the ongoing data collection using LimeSurvey Offline-App for android systems. Data control and consistency checks were carried out at monthly intervals and a simple plausibility check was carried out immediately after the entry of a maximum of 30 data sets. Data were stored in anonymous form on a computer at the University of Leipzig network in accordance with the data protection guidelines.

**Fig. 1:**
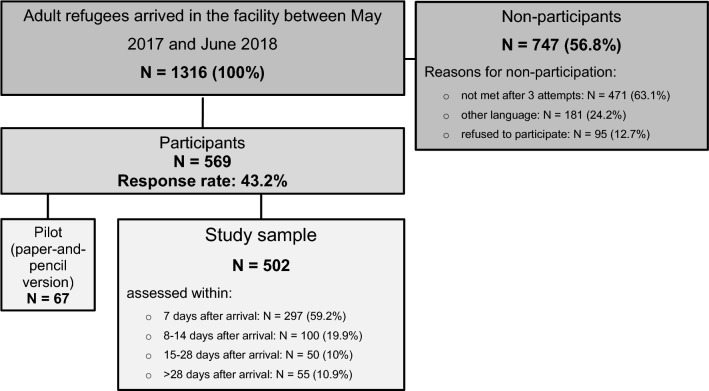
Study procedure; Leipzig (Germany), May 2017–June 2018

A total of 1316 adult individuals were newly accommodated in the primary reception facility during the survey period, 569 of whom took part in the study (response rate 43.2%). Of these, 67 individuals completed the paper version of the questionnaire and 502 responded via tablet. Generally, about 60% (*n* = 297) were assessed during the first seven days after the arrival, another 19.9% (*n* = 100) during the second week (between 8 and 14 days) after the arrival, 10% (*n* = 50) during the period of 15 and 28 days after the arrival, and finally 10.9% (*n* = 55) > 28 days after the arrival. Data on all non-participants’ age, sex, and country of origin were recorded to identify possible selection bias. Detailed information on age, sex and country of origin of non-participants as well as calculated non-response weights were published previously (Nesterko et al. 2020). Figure 1 gives an overview of the study procedure.

The study was approved by the Ethics Committee of the Medical Faculty of the University of Leipzig (446/16-ek). All study procedures were conducted in accordance with the Helsinki Declaration and its later amendments, or comparable ethical standards. Written informed consent was granted by all study participants.

### Measures

The questionnaire used in the present study included (1) socio-demographic, flight-related questions and questions addressing different social care needs, (2) standardized instruments for assessing PTSD, depression, and somatization, as well as (3) questions assessing self-rated mental and physical health, and current treatment needs for mental and physical health. The German version of the questionnaire was translated and back-translated into 10 different languages (Albanian, Arabic, English, Farsi, French, Kurdish, Russian, Spanish, Turkish and Urdu) by a professional translation agency (mt-g medical translation GmbH) specialized in medical translations. The Tigrinya version of the questionnaire was translated and back-translated by the same agency based on the English version of the questionnaire. All back-translations were reviewed by the first and last author and, when necessary, returned to the agency for final modification/adjustment.

#### Sociodemographic and flight-related characteristics as well as social care needs

Participants were asked to provide information about their age, sex, country of origin, marital status, number of children, level of education, the duration of their flight, accompaniment during their flight, as well as their present needs for support and/or assistance due to the asylum procedure, access to the labor market and education system, family reunion requests, assistance in navigating health care system, German language training, and/or private housing, each with “yes” or “no” response options. Finally, participants’ length of stay in the cooperating facility was assessed using registration data provided by the facility staff.

#### Symptoms of somatization, depression, and post-traumatic stress disorder

Somatic symptoms were assessed with the Somatic Symptom Scale-8 (SSS-8) (Gierk et al. 2014). The SSS-8 is a shortened version of the PHQ-15 questionnaire developed for DSM-5 field trials. Each item can be rated on a 5-point Likert-Scale from “not at all” (0) to “very much” (4) referring to the previous 7 days. The total scores, therefore, range from 0 to 32, and are subdivided into five categories of severity: “none to minimal” (0–3), “low” (4–7), “medium” (8–11), “high” (12–15), and “very high” (16–32) somatic symptom burden. A cut-off-score of > 11 was used for the present study. The internal consistency was *α* = 0.84 (0.77 to 0.93 for the different language versions).

Depression symptoms were assessed with the Patient Health Questionnare-9 (PHQ-9) (Kroenke et al. 2001). The PHQ-9 contains nine items rated on a scale of 0 (“not at all”) to 3 (“nearly every day”) which reflect the frequency with which participants have experienced the symptom in question within the previous 14 days. Based on the total sum (0–27), symptom severity can be divided into the categories “none-minimal” (0–4), “mild” (5–9), “moderate” (10–14), “moderately severe” (15–19), and “severe” (20–27) depression. Participants with a sum score of > 14 were classified as having depressive disorder. Cronbach’s *α* in the present study was *α* = 0.84 (0.70 to 0.89 for the different language versions).

PTSD was assessed with the PCL-5 (PTSD-Checklist), a 20-item self-report instrument, which assesses symptoms of PTSD as defined by the DSM-5 (Blevins et al. 2015). The 20 items of the PCL-5 reflect the frequency with which respondents have experienced the item in question rated on a 5-point Likert-scale ranging from “not at all” (0) to “extremely” (4). A total score (0–80) can be obtained by summing up the scores for each of the 20 items. A score at or above the cut-off score of 33 indicates the presence of PTSD in the respondent. Cronbach’s’ *α* in the present study was *α* = 0.95 (0.93 to 0.97. for the different language versions).

#### Traumatic events

Traumatic events were assessed using the revised DSM-5 (LEC-5) Life Events Checklist for assessing trauma exposure (Weathers et al. 2013). The LEC-5 is comprised of 16 items, which address types of events that can potentially result in PTSD or distress. The following response categories exist for each type of event: (1) happened to me, (2) witnessed it, (3) learned about it, (4) part of my job, (5) not sure, and (6) does not apply. The LEC-5 was used in combination with the PCL-5 (Posttraumatic stress disorder Checklist as defined by DSM-5) for the purpose of establishing exposure to a PTSD A-Criterion, with the response option “happened to me”, “witnessed it” and “part of my job” being used in the present study.

#### Self-rated mental and physical health status and current needs for treatment

**Fig. 2:**
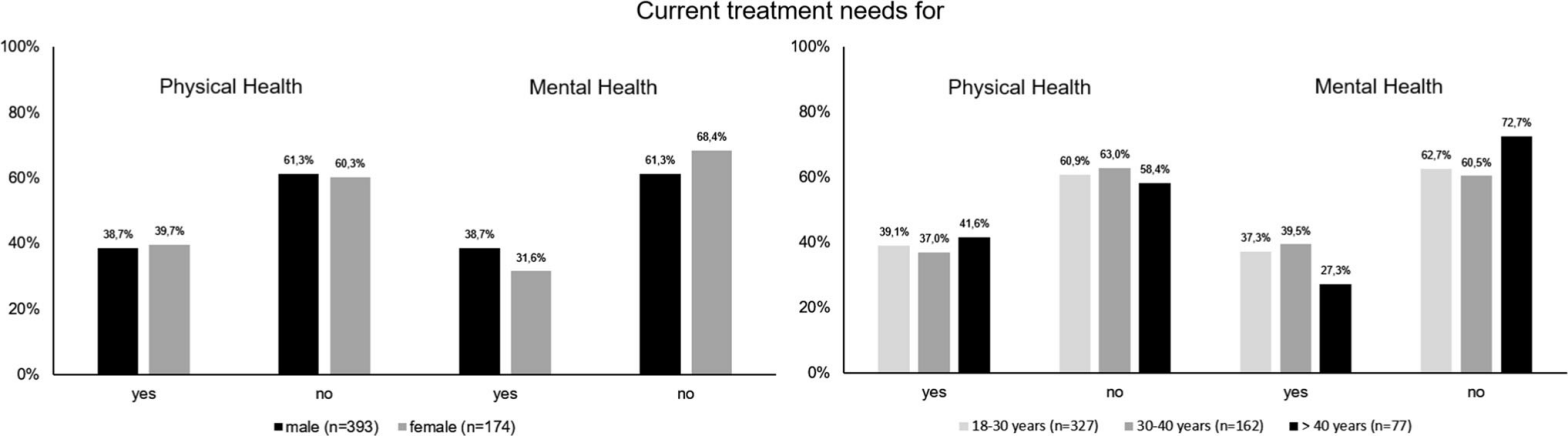
Current treatment needs for physical and mental health stratified by sex and age groups; Leipzig (Germany) May 2017–June 2018

Participants were asked to rate their current mental and physical health status on a visual analog scale ranging from 0 to 100, with higher scores indicating better health status. In addition, current treatment needs for mental and/or physical health were assessed separately using two items (“Do you currently need treatment for physical problems?”/“Do you currently need treatment for mental/emotional problems?”) with “yes” or “no” response options (Fig. 2).

For the purposes of the present study, focusing on newly arrived refugees’ mental and physical treatment needs, we made a distinction between participants who had no clinically significant symptoms of somatization, depression and PTSD, and those who screened positive for at least one of mental disorders investigated. Results on prevalence and comorbidity of somatization, depression and PTSD, as well as traumatic events experienced in participants of this sample are described in more detail previously (Nesterko et al. 2020).

### Statistical analyses

Statistical analyses were performed using the IBM SPSS statistical package, version 24.0 for Windows. Descriptive statistics including frequencies, means, and standard deviations were used to characterize the study sample. The symptom burden levels of the mental disorders investigated were calculated according to the cut-off scores of each questionnaire. Two logistic regression analyses were conducted using the Enter method—consisting of the same set of predictor variables (age, sex, university degree, partnership, parenthood, flight duration, accompaniment during flight, number of traumatic events, positive screening result for at least one mental disorder under consideration, self-rated mental and physical health, assessment time after arrival, need for assistance in respect to asylum procedure, labor market, education system, family reunion requests, navigating the health care system, German language training, and private housing)—to look for potential predictors among (1) socio-demographic, (2) flight and (3) health related factors as well as (4) participants’ social care needs on their current treatment needs for mental and physical health.

## Results

Table 1 gives an overview of the sample’s socio-demographic and flight-related characteristics. The mean age of the participants in the present study was 29.84 (SD = 9.13) years. The majority of the participants were male (*n* = 395, 69.4%). The largest groups were refugees from Cameroon (*n* = 92, 16.2%), Venezuela (*n* = 85, 14.9%), and Syria (*n* = 55, 9.7%); all in all, participants from over 30 different countries took part in the survey. Almost half of the participants (*n* = 277, 49%) reported having a university degree. A total of 325 (57.2%) participants were single, 35.7% (*n* = 203) were married, 5.1% (*n* = 29) divorced, and 1.9% (*n* = 11) widowed, with 212 (37.3%) participants reporting that they currently have a partner, and 222 (93.1%) that they have children. The mean flight duration was 1.9 years (SD = 3.1), reflecting a wide range spanning from 0 to 27 years, with 44.2% (*n* = 251) of the participants reporting that they had been alone while fleeing. More than two-thirds (*n* = 381, 68.7%) of the participants expressed needing assistance with respect to their asylum procedure, the most infrequently mentioned need being that of assistance due to a family reunion request (*n* = 145, 26.1%). Current treatment needs for physical and mental health were reported by 217 (39.2%) and 202 (36.5%) participants, respectively.

**Table 1:** Sociodemographic and flight-related characteristics of the study sample; study conducted in Leipzig (Germany) between May 2017 and June 2018

	Participants *N* = 569
Age^1^	
M/SD/Range	29.84/9.13/18–70
Sex	
Male	395 (69.4%)
Female	174 (30.6%)
Country of origin	
Cameroon	92 (16.2%)
Eritrea	49 (8.6%)
Iraq	28 (4.9%)
Nigeria	38 (6.7%)
Syria	55 (9.7%)
Turkey	52 (9.1%)
Venezuela	85 (14.9%)
Other^a^	170 (29.9%)
University degree^2^	
Yes	277 (49%)
No	288 (51%)
Marital status^1^	
Single	325 (57.2%)
Married	203 (35.7%)
Divorced	29 (5.1%)
Widowed	11 (1.9%)
Partnership^1^	
Yes	212 (37.3%)
No	356 (62.7%)
Parenthood^1^	
Yes	222 (39.1%)
No	346 (60.9%)
Flight duration in years^3^	
M/SD/Range	1.9/3.1/0–27
Accompaniment during the flight^1^	
Alone	251 (44.2%)
Strangers	141 (24.8%)
Friends	57 (10%)
Family members	119 (21%)
Social care needs due to:	
Finding work^4^	
Yes/no	307 (55.3%)/248 (44.7%)
Asylum procedure^4^	
Yes/no	381 (68.6%)/174 (31.4%)
Education^4^	
Yes/no	234 (42.2%)/321 (57.8%)
Family reunion^4^	
Yes/no	145 (26.1%)/410 (73.9%)
Navigating within HCS^b,4^	
Yes/no	217 (39.1%)/338 (60.9%)
German language training^4^	
Yes/no	354 (63.8%)/201 (36.2%)
Finding apartment^4^	
Yes/no	225 (40.5%)/330 (59.5%)
Current need for:	
Physical health care^5^	
Yes/no	217 (39.2%)/337 (60.8%)
Mental health care^5^	
Yes/no	202 (36.5%)/352 (63.5%)

^a^Country of origin other (*N*): Afghanistan (11), Algeria (4), Armenia (3), Belarus (1), Colombia (1) Ethiopia (20), Ghana (3), Georgia (9), India (2), Iran (7), Jordan (2), Kosovo (1), Kuwait (1), Lebanon (7), Liberia (1), Libya (25), Morocco (5), Myanmar (3), Palestine (13), Pakistan (7), Russian Federation (12), Senegal (2), Somalia (7), Sri Lanka (1), Tunisia (7), Ukraine (1), stateless (11)

^b^Health care system; ^1^*N* = 568; ^2^*N* = 566; ^3^*N* = 544; ^4^*N* = 555; ^5^*N* = 554

Prevalence rates of somatization, depression, and PTSD as well as mean scores for self-rated mental and physical health, both stratified by sex and in total, are displayed in Table 2. The highest prevalence rate was found for symptoms of PTSD (35.7%), followed by symptoms of somatization (31.3%), and depression (21.3%). The mean number of different traumatic events was 4.8 (SD = 3.7). All in all, half of the participants (*n* = 278, 50.1%) screened positive for at least one mental disorder. More details about prevalence and comorbidity of somatization, depression, and PTBS, as well as traumatic events experienced in participants from this sample are described elsewhere (Nesterko et al. 2020).

**Table 2:** Symptom burden of somatization, depression, and posttraumatic stress disorder as well as number of traumatic events and self-rated mental and physical health in recently arrived refugees; study conducted in Leipzig (Germany) between May 2017 and June 2018

	Female *N *(%)	Male *N *(%)	Total *N* (%)
Somatization^1^			
SSS-8 cut off > 11	77 (45)	97 (25)	174 (31.3)
Depression^2^			
PHQ-9 cut-off > 14	41 (23.7)	79 (20.3)	120 (21.3)
PTSD^3^			
PCL-5 cut-off > 32	66 (39.3)	133 (34.2)	199 (35.7)
Screened positive for at least one mental disorder investigated^4^	101 (59.8)	177 (45.9)	278 (50.1)

	Female M/SD	Male M/SD	Total M/SD
Number of traumatic events^5^			
Range 0–15	4.14/3.56	5.08/3.75	4.8/3.7
Self-rated Mental Health^6^			
Range 0–100	42.02/34.28	48.10/34.59	46.23/34.58
Self-rated Physical Health^7^			
Range 0–100	51.58/32.71	57.55/33.54	55.71/33.37

^1^*N* = 559 (female *n* = 171, male *n* = 388); ^2^*N* = 563 (female *n* = 173, male *n* = 390); ^3^*N* = 557 (female *n* = 168, male *n* = 389); ^4^*N* = 555 (female *n* = 169, male *n* = 386); ^5^N = 569 (female *n* = 174, male *n* = 395); ^6^*N* = 563 (female *n* = 173, male *n* = 390); ^7^*N* = 566 (female *n* = 174, male *n* = 392)

Two separate logistic regression analyses were performed to test which socio-demographic, flight-related, mental health-related factors, and social care needs are associated with current treatment needs for physical and mental health (Table 3). In both models, participants with a higher number of traumatic events (OR: 1.06, 95% CI: 1.00–1.13; OR: 1.07, 95% CI: 1.01–1.13), those who screened positive for at least one mental disorder (OR: 4.52, 95% CI: 2.76–7.40; OR: 2.73, 95% CI: 1.73–4.31), and participants who reported a current need for support with respect to the navigating the health care system (OR: 2.14, 95% CI: 1.21–3.77; OR: 3.25, 95% CI: 1.92–5.50) were found to be more likely to report current treatment needs for mental and physical health. Participants with worse self-rated physical health are more likely to have current treatment need for physical health (OR: 0.97, 95% CI: 0.96–0.98). Similarly, participants with worse mental health are more likely to state mental health treatment needs (OR: 0.98, 95% CI: 0.97–0.99). In addition, male participants were more likely than females to report current treatment needs for mental health problems (OR: 2.36, 95% CI: 1.39–4.02).

**Table 3:** Logistic regression analyses predicting mental and physical treatment needs in newly arrived refugees; study conducted in Leipzig (Germany) between May 2017 and June 2018

Predictor*	Current need for mental health care (*N* = 524)			Current need for physical health care (*N* = 524)		
	Adjusted OR	95% CI	*p*	Adjusted OR	95% CI	*p*
Age	0.99	0.96–1.02	0.434	1.01	0.98–1.04	0.454
Sex^1^	2.36	1.39–4.02	0.002	1.28	0.79–2.05	0.313
University degree^2^	1.03	0.64–1.65	0.906	1.03	0.67–1.59	0.890
Partnership^3^	1.00	0.61–1.64	0.999	1.01	0.64–1.59	0.974
Parenthood^4^	0.75	0.43–1.33	0.330	1.20	0.71–2.04	0.492
Flight duration	1.02	0.95–1.10	0.575	1.03	0.97–1.10	0.337
Accompaniment during the flight^5^	0.73	0.44–1.21	0.226	0.92	0.58–1.46	0.731
Number traumatic events	1.06	1.00–1.13	0.048	1.07	1.01–1.13	0.023
At least one MD investigated^6^	4.52	2.76–7.40	< 0.001	2.73	1.73–4.31	< 0.001
Self-rated mental health	0.98	0.97–0.99	< 0.001	1.01	1.00–1.02	0.146
Self-rated physical health	0.99	0.98–1.00	0.070	0.97	0.96–0.98	< 0.001
Time of assessment^7^	0.83	0.67–1.04	0.114	0.94	0.77–1.14	0.535
Social care needs due to						
Finding work^6^	1.26	0.74–2.14	0.385	0.79	0.48–1.31	0.369
Asylum procedure^6^	0.99	0.57–1.71	0.967	1.23	0.75–2.03	0.407
Education^6^	1.36	0.82–2.26	0.233	0.66	0.41–1.07	0.093
Family reunion^6^	0.75	0.42–1.35	0.332	0.86	0.50–1.47	0.583
Health care system^6^	2.14	1.21–3.77	0.009	3.25	1.92–5.50	< 0.001
German language training^6^	1.30	0.75–2.25	0.356	1.07	0.65–1.77	0.797
Finding apartment^6^	0.59	0.34–1.05	0.074	0.64	0.38–1.10	0.107
Model fit indices						
^ χ2^/df/*p*	201.49/19/ < 0.001			137.99/19/ < 0.001		
− 2 Log-Likelihood	482.549			559.760		
Nagelkerkes *R*^2^	0.436			0.315		
Cox and Snell *R*^2^	0.319			0.232		

^1^Female = 1, male = 2; ^2^yes = 1, no = 2; ^3^partnership = 1, no partnership = 2; ^4^children = 1, no children = 2; ^5^unaccompanied = 1, accompanied = 2; ^6^yes = 1, no = 0; ^7^within first 7 days after arrival = 1, between 8 and 14 days = 2, between 15 and 28 days = 3, after 28 days = 4

*The lower category represents the reference category

*MD* mental disorder

## Discussion

In the present study, socio-demographic and flight-related characteristics, as well as mental health problems and social care needs were analyzed as predictors for self-rated current needs in treatment for physical and mental health problems among newly arrived refugees in Germany. Participants who: reported a higher number of traumatic events, screened positive for at least one mental disorder, and/or had a current need for support navigating the health care system were more likely to report current treatment needs for mental and physical health. In addition, worse self-rated physical and mental health, were found to be predictors for physical and mental health treatment needs. A rather unexpected association was found between mental health treatment needs and sex, whereby male participants were found to report a greater need for treatment of their mental health. In contrast to our findings, previous studies have frequently observed higher symptom burden, suggesting higher rates of health care needs, and in consequence, higher health care utilization among female refugees (Yang and Hwang 2016). When examining the existing evidence on mental health care needs, no conclusive statement on sex differences can be made; there are some studies that report more use (Kerkenaar et al. 2013; Nielsen et al. 2015) and some that indicate less use of mental health services by female immigrants and refugees in comparison to their male counterparts (Durbin et al. 2014, 2015; Straiton et al. 2014, 2016). In a population-based, cross-sectional study examining access to health care among asylum-seekers in Germany (Bozorgmehr et al. 2015), higher rates of health care utilization in female participants were partly explained by greater medical needs in women with respect to pregnancy and childbirth. Moreover, there is some evidence suggesting cultural and/or ethnic differences resulting in varying perceptions of mental health problems and consequently mental health care needs across different groups of immigrants and refugees (Kohrt et al. 2014; Nesterko et al. 2017). However, by controlling self-rated health status and different mental health outcomes of the ethnically quite heterogeneous study sample we found current treatment needs for mental, but not physical health problems differing between men and women, revealing higher rates by males. One possible explanation might be related to the higher number of traumatic events reported by men. Future research into this aspect is warranted (e.g., exposure to war-related traumatic events like captivity or torture leading to higher treatment needs). It should investigate how sex differences are associated with mental health treatment needs in refugees by considering possible impacts of both ethnic characteristics (e.g., analyses on more homogeneous groups) as well as specific flight-related traumatic exposure in male and female refugees.

In summary, with the exception of the sex differences in current treatment needs for mental health discussed above, health-related rather than socio-demographics, flight-related characteristics and social care needs were found to be associated with current treatment needs for mental and physical health problems among the participants in the present study.

Although the present study has some major strengths—(1) epidemiological approach, (2) assessment of recently arrived refugees, considering the time frame of symptom burden and excluding long-term post-migration stressors, (3) use of instruments that have been translated and back-translated into 11 different languages and (4) assessment of self-rated physical and mental health status, as well as needs due to numerous social requests right after arrival, something that has not been investigated in a comparable population before—there is a number of limitations requiring some critical reflection. First, the analyses conducted in the present study are based on cross-sectional data. Consequently, the generalizability of the findings is somewhat restricted due to the fact that (1) the data reflect a specific wave of refugees who had recently arrived in Germany at the time of data collection (e.g., refugees from Venezuela, Cameroon, and Syria) and (2) no information can be derived with respect to the long-term impact of the predictors analyzed. In addition, no conclusions can be drawn concerning the trajectories of subjective needs among this study’s participants or their health care utilization. Thus, future research should focus on refugee’s health care and treatment needs and health care utilization using a longitudinal approach. Due to the methodological approach used in the present study, the examination of health care utilization in recently arrived refuges was not possible, something which should be addressed in future research as well. Moreover, future research focusing on differences in mental health outcomes due to different cultural and/or ethnic affiliations of the participants as well as with respect to possible measurement invariance across different language versions of instruments is needed (Dere et al. 2015; Haroz et al. 2016; Kohrt et al. 2014; Schnyder et al. 2015). Third, due to the heterogeneity of refugee populations, both with regard to their countries of origin as well as with regard to different health care systems provided by the receiving countries, an international collaborative study on health care in refugee populations should be initiated by researchers in different receiving countries. Beyond that, in the vein of theoretical models on health service utilization in general populations (e.g., Andersen’s health behavior model; Andersen 1995), there is an urgent need for a specific theoretical framework explaining health care utilization in refugees, although some first suggestions addressing immigrant populations are already available (Yang and Hwang 2016).

Despite the limitations and implications for future research mentioned above, the results of the present study provide first insights into health care and treatment needs in newly arrived refugees for both academia and policy makers to, on the one hand, identify those in need, and on the other, to provide appropriate health care as soon as possible. In contrast to some debates in politics and the media arguing for more restrictions on asylum procedures—including costs and qualifying conditions for health care—with respect to would-be financial claims refugees are looking for, no such relation between different requests or rather different social and health care needs just after their arrival were found. What is more, there is research indicating that higher costs are imposed on the health care system as result of this population underutilizing health care services in general, and consequently over-utilizing emergency health services (Yang and Hwang 2016; Bauhoff and Göpffarth 2018), the method of treatment delivery provided for by the Asylum-Seekers’ Benefits Act (AsylbLG). In conclusion, from our point of view, providing appropriate health care for refugees in need, a humanitarian obligation that western high-income countries must not shirk, is hardly possible without appropriate policy changes enacted by politically responsible representatives.

## Abbreviations

AsylbLG: Asylum-Seekers’ Benefits Act
PTSD: Posttraumatic stress disorder
UNHCR: United Nations High Commissioner for Refugees

## References

[CR1] Andersen RM (1995). Revisiting the behavioral model and access to medical care: does it matter?. J Health Soc Behav.

[CR2] Bauhoff S, Göpffarth D (2018). Asylum-seekers in Germany differ from regularly insured in their morbidity, utilizations and costs of care. PLoS ONE.

[CR3] Biddle L, Menold N, Bentner M, Nöst S, Jahn R, Ziegler S, Bozorgmehr K (2019). Health monitoring among asylum seekers and refugees: a state-wide, cross-sectional, population-based study in Germany. Emerg Themes Epidemiol.

[CR4] Bischoff A, Denhaerynck K (2010). What do language barriers cost? An exploratory study among asylum seekers in Switzerland. BMC Health Serv Res.

[CR5] Blevins CA, Weathers FW, Davis MT, Witte TK, Domino JL (2015). The posttraumatic stress disorder checklist for DSM-5 (PCL-5): development and initial psychometric evaluation. J Trauma Stress.

[CR6] Bogic M, Njoku A, Priebe S (2015). Long-term mental health of war-refugees: a systematic literature review. BMC Int Health Hum Rights.

[CR7] Borgschulte HS, Wiesmüller GA, Bunte A, Neuhann F (2018). Health care provision for refugees in Germany - one-year evaluation of an outpatient clinic in an urban emergency accommodation. BMC Health Serv Res.

[CR8] Bozorgmehr K, Schneider C, Joos S (2015). Equity in access to health care among asylum seekers in Germany: evidence from an exploratory population-based cross-sectional study. BMC Health Serv Res.

[CR9] Dere J, Watters CA, Yu SC-M, Bagby RM, Ryder AG, Harkness KL (2015). Cross-cultural examination of measurement invariance of the Beck Depression Inventory-II. Psychol Assess.

[CR10] Durbin A, Lin E, Moineddin R, Steele LS, Glazier RH (2014). Use of mental health care for nonpsychotic conditions by immigrants in different admission classes and by refugees in Ontario, Canada. Open Med.

[CR11] Durbin A, Moineddin R, Lin E, Steele LS, Glazier RH (2015). Mental health service use by recent immigrants from different world regions and by non-immigrants in Ontario, Canada: a cross-sectional study. BMC Health Serv Res.

[CR12] Fazel M, Wheeler J, Danesh J (2005). Prevalence of serious mental disorder in 7000 refugees resettled in western countries: a systematic review. Lancet.

[CR13] Gerritsen AAM, Bramsen I, Devillé W, van Willigen LHM, Hovens JE, van der Ploeg HM (2006). Use of health care services by Afghan, Iranian, and Somali refugees and asylum seekers living in The Netherlands. Eur J Public Health.

[CR14] Giacco D, Laxhman N, Priebe S (2018). Prevalence of and risk factors for mental disorders in refugees. Semin Cell Dev Biol.

[CR15] Gierk B, Kohlmann S, Kroenke K, Spangenberg L, Zenger M, Brähler E, Löwe B (2014). The somatic symptom scale-8 (SSS-8): a brief measure of somatic symptom burden. JAMA Intern Med.

[CR16] Goosen ESM (2014) A safe and healthy future? Epidemiological studies on the health of asylum seekers and refugees in the Netherlands. PhD Thesis, Avaliable at: https://pure.uva.nl/ws/files/2041751/141318_thesis.pdf. Accessed 17 June 2020

[CR17] Hadgkiss EJ, Renzaho AMN (2014). The physical health status, service utilisation and barriers to accessing care for asylum seekers residing in the community: a systematic review of the literature. Aust Health Rev.

[CR18] Haroz EE, Bolton P, Gross A, Chan KS, Michalopoulos L, Bass J (2016). Depression symptoms across cultures: an IRT analysis of standard depression symptoms using data from eight countries. Soc Psychiatry Psychiatr Epidemiol.

[CR19] Henjum S, Barikmo I, Strand TA, Oshaug A, Torheim LE (2012). Iodine-induced goitre and high prevalence of anaemia among Saharawi refugee women. Public Health Nutr.

[CR20] Kerkenaar MME, Maier M, Kutalek R, Lagro-Janssen ALM, Ristl R, Pichlhöfer O (2013). Depression and anxiety among migrants in Austria: a population based study of prevalence and utilization of health care services. J Affect Disord.

[CR21] Kiss V, Pim C, Hemmelgarn BR, Quan H (2013). Building knowledge about health services utilization by refugees. J Immigr Minor Health.

[CR22] Kohrt BA, Rasmussen A, Kaiser BN, Haroz EE, Maharjan SM, Mutamba BB, de Jong JTVM, Hinton DE (2014). Cultural concepts of distress and psychiatric disorders: literature review and research recommendations for global mental health epidemiology. Int J Epidemiol.

[CR40] Kroenke K, Spitzer RL, Williams JB (2001). The PHQ-9: validity of a brief depression severity measure. J Gen Intern Med.

[CR23] Medical care for refugees e.V. (2020) Electronic Health Cards for Asylum Seekers in Germany. https://gesundheit-gefluechtete.info/gesundheitskarte/. Accessed 8 Apr 2020

[CR24] Nesterko Y, Kaiser M, Glaesmer H (2017). Kultursensible Aspekte während der Diagnostik von psychischen Belastungen bei Flüchtlingen—Zwei kommentierte Fallberichte (Culture-Sensitive Aspects in Diagnostics of Mental Distress in Refugees - Two Commented Case Reports). Psychother Psychosom Med Psychol.

[CR25] Nesterko Y, Turrión CM, Friedrich M, Glaesmer H (2019). Trajectories of health-related quality of life in immigrants and non-immigrants in Germany: a population-based longitudinal study. Int J Public Health.

[CR26] Nesterko Y, Jäckle D, Friedrich M, Holzapfel L, Glaesmer H (2020). Prevalence of post-traumatic stress disorder, depression and somatisation in recently arrived refugees in Germany: an epidemiological study. Epidemiol Psychiatr Sci.

[CR27] Newbold B (2009). The short-term health of Canada's new immigrant arrivals: evidence from LSIC. Ethn Health.

[CR28] Nielsen SS, Jensen NK, Kreiner S, Norredam M, Krasnik A (2015). Utilisation of psychiatrists and psychologists in private practice among non-Western labour immigrants, immigrants from refugee-generating countries and ethnic Danes: the role of mental health status. Soc Psychiatry Psychiatr Epidemiol.

[CR29] Sarría-Santamera A, Hijas-Gómez AI, Carmona R, Gimeno-Feliú LA (2016). A systematic review of the use of health services by immigrants and native populations. Public Health Rev.

[CR30] Schnyder U, Müller J, Morina N, Schick M, Bryant RA, Nickerson A (2015). A comparison of DSM-5 and DSM-IV diagnostic criteria for posttraumatic stress disorder in traumatized refugees. J Trauma Stress.

[CR31] Straiton M, Reneflot A, Diaz E (2014). Immigrants' use of primary health care services for mental health problems. BMC Health Serv Res.

[CR32] Straiton ML, Powell K, Reneflot A, Diaz E (2016). Managing mental health problems among immigrant women attending primary health care services. Health Care Women Int.

[CR33] United Nations High Commissioner for Refugees (2019) Global Trends. Forced Displacement in 2018. https://www.unhcr.org/5d08d7ee7.pdf

[CR34] Wångdahl J, Lytsy P, Mårtensson L, Westerling R (2018). Poor health and refraining from seeking healthcare are associated with comprehensive health literacy among refugees: a Swedish cross-sectional study. Int J Public Health.

[CR35] Weathers FW, Blake DD, Schnurr PP, Kaloupek DG, Marx BP, Keane TM (2013) The Life Events Checklist for DSM-5 (LEC-5). www.ptsd.va.gov

[CR36] Wetzke M, Happle C, Vakilzadeh A, Ernst D, Sogkas G, Schmidt RE, Behrens GMN, Dopfer C, Jablonka A (2018). Healthcare utilization in a large cohort of asylum seekers entering Western Europe in 2015. Int J Environ Res Public Health.

[CR37] World Health Organization (2018) Report on the health of refugees and migrants in the WHO European Region: No PUBLIC HEALTH without REFUGEE and MIGRANT HEALTH. https://www.euro.who.int/__data/assets/pdf_file/0005/392774/ermh-summary-eng.pdf?ua=1.

[CR38] Yang PQ, Hwang SH (2016). Explaining immigrant health service utilization. SAGE Open.

[CR39] Yun K, Hebrank K, Graber LK, Sullivan M-C, Chen I, Gupta J (2012). High prevalence of chronic non-communicable conditions among adult refugees: implications for practice and policy. J Community Health.

